# Burden of disease of seven dietary risk factors in population groups with similar lifestyle patterns in Denmark

**DOI:** 10.1007/s00394-025-03715-9

**Published:** 2025-06-02

**Authors:** Sayantan Sengupta, Sofie T. Thomsen, Aleksandra Davydova, Hernan G. Redondo, Lea S. Jakobsen, Sara M. Pires

**Affiliations:** Risk-Benefit Research Group, National Food Institute, Technical University of Denmark, Lyngby, 2800 Denmark

**Keywords:** Burden of disease, Diets, Dietary risk factors, Clusters, DALYs

## Abstract

**Purpose:**

Identifying subgroups of the population with similar diet and lifestyle characteristics is useful to identify the most important dietary risk factors within these groups and thus provide evidence for targeted interventions. The aim of this study was to estimate the burden of disease of dietary risk factors in the population groups with similar lifestyles in Denmark.

**Methods:**

We applied a comparative risk assessment approach to estimate the burden of disease attributable to exposure to seven risk factors associated with six health outcomes, selected through a review of the literature. We collected national dietary intake data, dose-response associations, and disease burden of selected health outcomes. Individuals were grouped into 12 clusters with similar lifestyles and dietary patterns. The burden of disease was evaluated in terms of disability adjusted life year (DALY) change of shifting from the current intake to recommended intakes. We applied Monte Carlo simulation to quantify associated uncertainties.

**Results:**

The total disease burden attributable to the seven dietary risk factors varied substantially across clusters, between 14 and 3332 DALYs/100,000 inhabitants. Cardiovascular disease was the leading health outcome across all clusters, and the major risk factors contributing to the burden were low consumption of tree nuts and whole grains. Clusters with older adults, higher BMI, and lower physical activity had the highest burden, and leading risk factors were low consumption of tree nuts, whole grains and vegetables. Urban, educated, and physically active individuals had the lowest disease burden.

**Conclusion:**

Our results showed that the burden of sub-optimal diets is unevenly distributed across population groups that have similar characteristics in terms of diet, lifestyle and socioeconomics. Our estimates on the ranking of dietary risk factors within each population subgroup can be used to tailor public health intervention strategies.

**Supplementary Information:**

The online version contains supplementary material available at 10.1007/s00394-025-03715-9.

## Introduction

Unhealthy diets are a leading cause of disease globally. Dietary patterns with too high consumption of meat and meat products, saturated fat, and salt, and low consumption of for example fruits and vegetables, legumes, and fish are associated with an increased risk of several non-communicable diseases (NCDs) [1]. The Global Burden of Disease (GBD) study estimated that 15 dietary risk factors led to the loss of 178 million healthy years globally and approximately 95,000 in Denmark in 2021 [2] The GBD estimates for Denmark showed that the disease burden attributable to dietary risk factors was highest for males above 70 years (when adjusted per population size) [3]. However, while stratifying burden of disease by age and sex is informative, these categories are comprised of people with potentially very distinct diets, lifestyle, and socioeconomic characteristics, all important determinants of health. Knowledge on the influence of these factors on the distribution of burden of disease across the population is also crucial to inform public health strategies that would be more efficient in reducing the burden of disease in specific population groups.

The associations between lifestyle, socioeconomics and dietary patterns have been previously investigated in Denmark, both at the whole population level and by subgrouping the population based on food consumption patterns. Groth et al. 2014 [4] examined associations between dietary habits, alcohol habits, physical activity, and socioeconomic factors in Danish adults based on the Danish National Survey of Diet and Physical Activity (DANSDA) from 1995 to 2008 and found that both diet quality and physical inactivity differed systematically with educational group, with healthier habits in the group with long education and dietary and alcohol habits furthest away from the guidelines in men. Also based on DANSDA data from adults (2005 to 2008), Knudsen et al. explored dietary patterns through principal component analysis and investigated the associations with various demographic and lifestyle factors (age, gender, weight, height, physical activity, smoking habits, educational level, and attitudes towards healthy eating) [5]. This study identified three major dietary patterns (‘traditional’; ‘health-conscious’; and ‘fast food’ patterns) and found that the ‘traditional’ pattern was positively associated with being male and increasing age, the ‘health-conscious’ pattern with being female, increasing age, and educational level; and the ‘fast food’ pattern was inversely associated with age and smoking. Both these studies concluded that their findings were valuable in addressing disparities, highlighting the need for more focused strategies in communicating food-based dietary guidelines. However, both studies did not demonstrate how these differences in dietary patterns across population groups reflect in actual public health impact. Importantly, they did not quantify the magnitude of the burden of disease attributable to current diets, nor how this burden differs between different population groups.

More recently, Herrera et al. applied a machine learning approach to identify subgroups in the Danish population similar in terms dietary preferences, lifestyle habits, and socioeconomic factors [6]. This study used more recent data from the same survey (2011–2013) and grouped participants into 12 clusters with distinct diet, lifestyle, and socioeconomic characteristics. Applying a burden of disease approach, the authors estimated the number of disability-adjusted life years (DALYs), a metric of healthy life years lost, due to dietary exposure to three chemicals commonly present in foods (methylmercury, inorganic arsenic, and cadmium) in those clusters. The estimated disease burden differed between subgroups, with those bearing the highest burden attributable to the chemicals generally with better adherence to dietary guidelines (i.e. high consumption of fish and seafood, fruits and vegetables, and cereals), higher education and physical activity levels, and being largely non-smokers. Identifying subgroups of the population with similar diet and lifestyle characteristics proved useful to identify the most important risk factors and social determinants within these groups and thus provide evidence for targeted interventions [6].

The aim of this study was to estimate the burden of disease of seven dietary risk factors (low consumption of tree nuts and peanuts, legumes, fish, dairy, whole grain, vegetables, and high consumption of red meat) in the 12 subgroups of the Danish population identified by Herrera et al. [6]. We estimated the burden in terms of DALYs, accounting for the risk of various NCDs associated with the risk factors. Our results will provide evidence for formulating targeted public health strategies aiming to promote shifts towards healthier diets.

## Methods

### Overview of the approach

We applied a comparative risk assessment approach to estimate the burden of disease that is attributable to exposure to each risk factor. We selected dietary risk factors that relate to consumption of foods, excluding single nutrients (i.e. saturated fat, sodium etc.), for which the national food based dietary guidelines recommend an intake [7]. The identification of health outcomes associated with the risk factors was based on a review of scientific evidence. We estimated the proportion of the total burden of disease of each health outcome in each population group prevented if exposure was changed to the theoretical minimum risk exposure level (TMREL). The TMREL was defined as the daily intake recommended by the Danish official dietary guidelines for all food groups except for red meat [7].

### Data

Food consumption data were available from the Danish Survey on Diet and Physical Activity (DANSDA) 2011-13 (DANSDA), a nation-wide, cross-sectional survey of diet and physical activity in a representative sample of the Danish population randomly selected from the civil registration system who filled out pre-coded semi-closed 7-day food diaries consisting of categories with common foods and dishes in the Danish diet [8, 9]. Herrera et al. identified population clusters based on DANSDA using the pre-processing and clustering capabilities implemented in the Compass method [10], a machine learning approach that uses non-linear associations among the variables as a basis to divide the subgroups in a population. All details of the approach can be found in [6]. We retrieved the participant identification numbers for the individuals in each cluster (personal communication) and extracted DANSDA consumption data for those individuals for the foods relevant to the following seven risk factors: too low consumption of whole grain; too low consumption of vegetables; too low consumption of legumes; too low consumption of peanuts and tree nuts; too low consumption of dairy; too low consumption of fish; and too high consumption of red meat.

### DALY envelopes

We retrieved disability adjusted life years (DALY) for the selected health outcomes for the Danish population 2019 from the GBD Results Tool [3]. We extracted DALYs per 100,000 Danish population stratified by sex and age groups defined by 5-year intervals (henceforth called “DALY envelopes”). For all health outcomes except colorectal cancer, we extracted DALY in ages greater than 15 years of age. For colorectal cancer, we extracted data for the age groups greater than 10 years of age.

### Selection of health effects and dose-response relationships

To identify the health outcomes associated with each dietary risk factor, we reviewed the reference lists of the GBD study [1], of the study by Schwingshackl et al. [11], and of World Cancer Research Fund reports [12, 13]. From these reference lists, we only considered systematic reviews that included meta-analyses of dose-response relationships between exposure to one of the seven food groups included in our assessment and the health outcomes associated with this exposure. We selected only those pairs for which the level of evidence was graded at least as “probable” as reported by the systematic reviews. When several systematic reviews provided dose-response data for the same risk factor-health outcome pair, we selected the most recent and with the highest number of studies analyzed. The selected dietary risk factor-health outcome pairs and the relative risks (RR) used to estimate the exposure-response functions for each of those pairs are shown in Table 1.

We applied the official dietary guidelines to define the TMREL applied to food groups, except for red meat. Instead of applying the recommended maximum consumption of meat, which includes to all types of meat (beef, pork, poultry, and lamb)), we used the consumption amount for red meat alone as was defined by Lassen and colleagues in a Danish-adapted healthy plant-based diet [14].

**Table 1:** Selected dietary risk factors, associated health outcomes, relative risks (RR), and theoretical minimum risk exposure levels (TMREL). T2D - type 2 diabetes, CRC - colorectal cancer, CC - colon cancer, IHD - ischemic heart disease, CVD– cardiovascular disease, M-males, F-females

Risk factor	Health outcome	Direction of the association	Dose- response relation	Overall intake range	RR	Reference	TMREL (g/day)
Too low consumption of peanuts and tree nuts	CVD	Inverse	Non-linear	0–25 g/day	0.79 (0.70;0.88) per 28 g/day	[15]	30
	IHD	Inverse	Non-linear	0–28 g/day	0.71 (0.63; 0.80) per 28 g/day	[15]	30
Too low consumption of legumes	IHD	Inverse	Non-linear	0–230 g/day	0.96 (0.92; 1.01) per 50 g/day	[16]	100
Too high consumption of red meat	CC	Positive	Non-linear	0–100 g/day	1.22 (1.06; 1.39) MW; 1.14 (0.82; 1.60) W; 1.07 (0.74; 1.56) M per 100 g/day	[17]	15
	CRC	Positive	Non-linear	0–100 g/day	1.12 (1.00; 1.25) MW; 1.02 (0.78; 1.33) W; 1.28 (0.49; 3.34) M; per 100 g/day	[17]	15
	T2D	Positive	Linear	1–170 g/day	1.17 (1.08; 1.26) per 100 g/day	[18]	15
Too low consumption of fish	IHD	Inverse	Non-linear	0–320 g/day	0.88 (0.79; 0.99) per 100 g/day	[16]	50
	Stroke	Inverse	Linear	0–2000 g/week	0.86 (0.75; 0.99) per 100 g/day	[16]	50
Too low consumption of dairy	T2D	Inverse	Non-linear	0–600 g/day	0.93 (0.87; 0.99) per 400 g/day	[19]	250
	Hypertension	Inverse	Linear	0–800 g/day	0.95 (0.94; 0.97) per 200 g/day	[20]	250
	CRC	Inverse	Non-linear	0–900 g/day	0.87 (0.83; 0.90)	[21]	250
Too low consumption of whole grain	T2D	Inverse	Non-linear	0–100 g/day	0.87 (0.82;0.93) per 30 g/day	[18]	75
	CRC	Inverse	Linear	0–374 g/day	0.95 (0.93;0.97) per 30 g/day	[22]	75
	IHD	Inverse	Non-linear	0–225 g/day	0.81 (0.75;0.87) per 90 g/day	[23]	75
	CVD	Inverse	Non-linear	0–210 g/day	0.78 (0.73;0,85) per 90 g/day	[23]	75
Too low consumption of vegetables	Stroke	Inverse	Non-linear	0–500 g/day	0.87 (0.79; 0.96) per 200 g/day	[24]	300
	CRC	Inverse	Non-linear	0–480 g/day	0.97 (0.96; 0.98) per 100 g/day	[22]	300
	IHD	Inverse	Non-linear	0–600 g/day	0.84 (0.79; 0.90) per 200 g/day	[24]	300

*The TMREL for the red meat group was defined based on Danish-adapted healthy plant-based diet that was developed by Lassen et al., 2020; TRMLs for the rest of the food groups were defined based on daily intakes of the food groups recommended by Official Danish Dietary Guidelines

### Model

We estimated the burden of disease of each dietary risk factor in four main steps. First, we estimated the mean consumption of each food group in each of the 12 population clusters. Second, the relative risk corresponding to the mean intake and the TMREL was estimated for each food group for all clusters. The relative risk was estimated from the dose-response meta data (i.e., RR per g increment of food) (Table 1). We assumed a log-linear association between relative risk and exposure and an RR of 1 at zero exposure. We did not extrapolate RRs outside the “overall intake range” observed in the studies (Table 1). Besides, if a non-linear relationship was visible in the studies, we assumed a log-linear relationship until the specific infliction point after which the RR levels out and thus no change in RR was modelled. In the third step, a population attributable fraction (PAF) for each dietary risk factor-health outcome pair was calculated using the relative risk at estimate mean intake for each cluster and recommended intake (i.e., TMREL). In the fourth step, we estimated the burden of disease for each dietary risk factor-health outcome pair by multiplying the DALY envelope for the specific health outcome for each cluster with the calculated PAF for the dietary risk factor-health outcome pair. When several food groups were risk factors for the same health outcome, we multiplied the PAFs for each risk factor-health outcome pair to estimate combined PAFs for the health outcome (Table 2). Because clusters were comprised of individuals of mixed age and gender, we assigned each individual in a cluster to the DALY envelope specific to that individual’s age and gender. The DALY envelopes assigned to the individuals were averaged to estimate the mean DALY envelope of each cluster.

We propagated uncertainty using Monte Carlo simulation, sampling from probability distributions fitted to the available data. Uncertainty originated from three variables: food consumption (uncertainty in mean daily consumption), relative risk, and DALY envelope. Normal distribution functions were used to model food consumption and DALY and a log-normal distribution for relative risk. For food consumption, empirical data were used to estimate the best fit for the population distribution of mean consumption (for each cluster); for relative risk and DALY, the reported mean and lower and upper bounds of the 95% confidence interval were used for distribution parameters (Table 2).

**Fig. 1:**
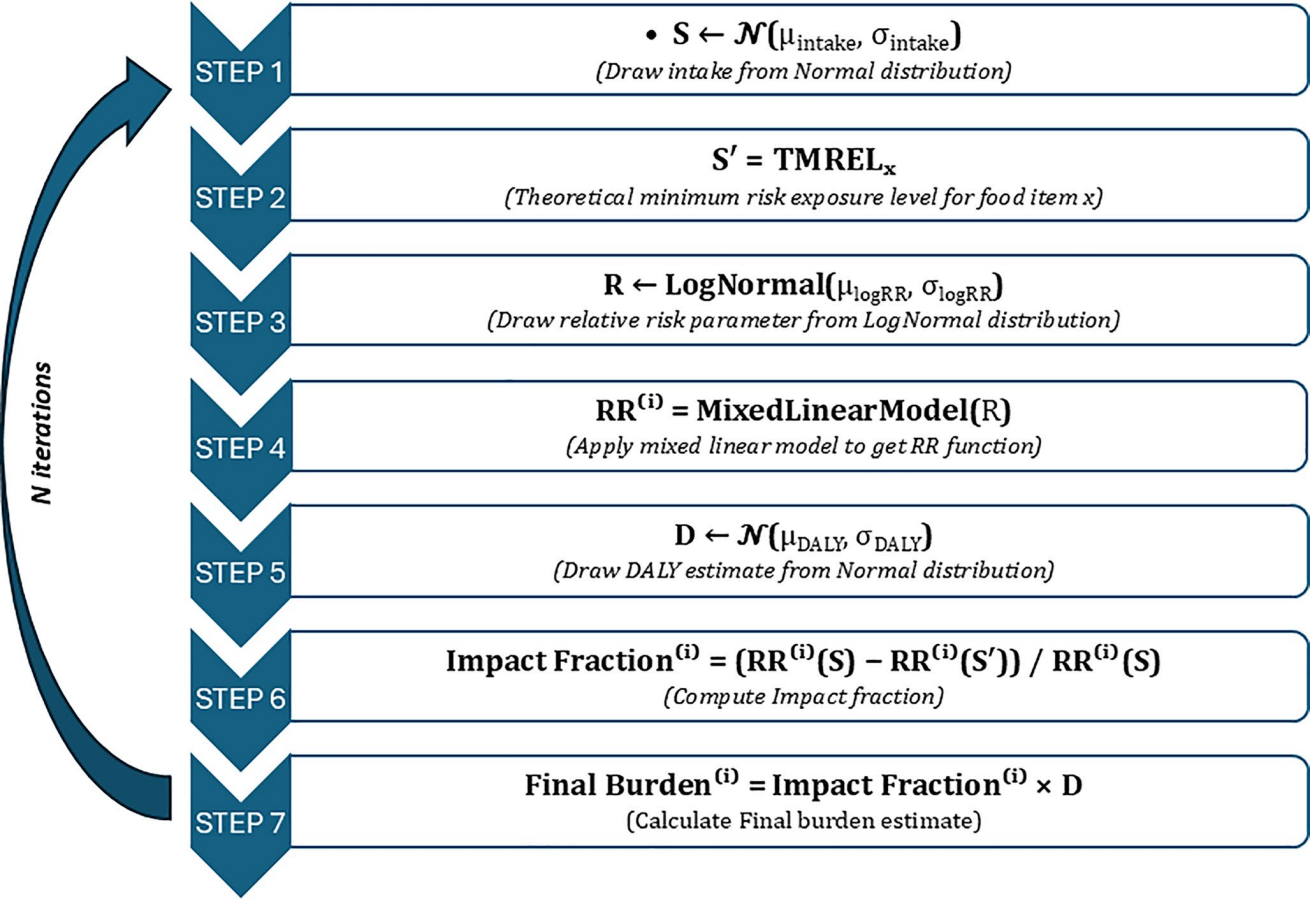
Flowchart of probabilistic burden estimation

10,000 samples were randomly sampled from the fitted distributions to estimate population distribution of DALY per 100,000 for each exposure-outcome pair in each cluster. The analysis was performed in Python programming language (Version 3.6) using scientific packages Pandas for data handling and SciPy for probability distribution functions. The pipeline for *n* iterations of the algorithm Probabilistic estimation of burden of a food item *X* is shown in Fig. 1. As a measure of convergence, standard error was used to monitor the dispersion of the sampling distribution (distribution of the mean DALYs for each risk factor and cluster) for the fixed number of iterations.

**Table 2:** Variables and distributions used to model the three sources of uncertainty. DALY: disability adjusted life year

Variable	Description	Equation	Distribution	Unit
Mean food intake of food groups per cluster	Mean amount of food consumed daily by individual *i* in the population	∑_i=1to n_ intake_(i)_	Normal (mean, standard deviation)	g/day
Dose response function for food j and health outcome z	Relative Risk of dietary risk factor j - health outcome z	ln(RR)=βx	Lognormal (log mean, log standard deviation)	Relative Risk
Population attributable fraction (PAF)	proportion of incidents in the population that are attributable to the risk factor.	${\matrix{ RR\left( {meanintake} \right) - \hfill \cr RR\left( {TMREL} \right) \hfill \cr} \over {RR\left( {TMREL} \right)}}$	Not Applicable (NA)	Between 0 and 1.
Combined PAF per health outcome	Combination of PAF health outcomes with several dietary risk factors	$\:{PAF}_{combined}=\:$ $\:1-\prod\:_{Risk\:Factors}(1-PAF)$	NA	Between 0 and 1.
DALY envelope for each health outcome	Upper and the lower bounds of each age group for health outcome z and sex t along with the mean.	DALY = Years of life lost + Years of life in Disability	Normal (mean, standard deviation)	DALY per 100,000 individuals.
Attributable burden	Health outcome attributable to a risk factor.	PAF × DALY envelope	NA	DALY per 100,000 individuals.

## Results

### Burden of disease attributable to dietary risk factors

**Fig. 2:**
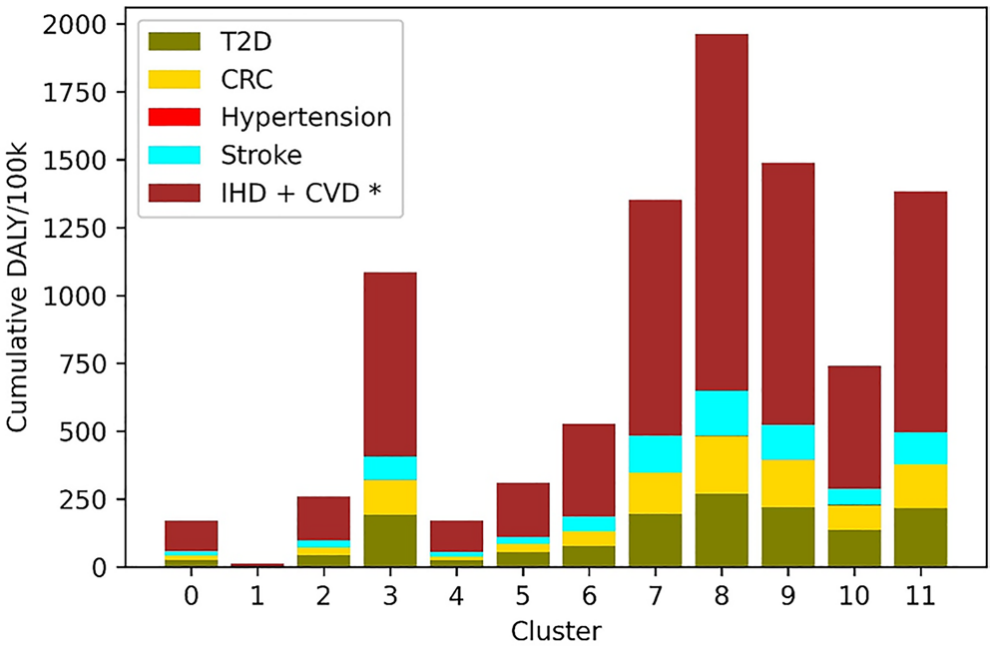
Burden of disease attributable to six health outcomes associated with seven dietary risk factors in 12 clusters of the Danish population (disability adjusted life years (DALYs) per 100,000 inhabitants. *T2D - type 2 diabetes*,* CRC - colorectal cancer*,* IHD - ischemic heart disease*,* CVD– cardiovascular disease.* *CVD and IHD, respectively, are in this figure added up for each cluster but are associated with different risk factors in a given cluster

The total disease burden attributable to the seven dietary risk factors varied substantially across clusters, between 14 and 3332 DALYs/100,000 inhabitants (Fig. 2). Results showed that the clusters experiencing lower burden of disease were clusters 0, 1 and 4, whereas others experienced a burden up to 220 times higher, especially cluster 8. Cardiovascular disease, including ischemic heart disease, was the health outcome with the largest estimated disease burden in all clusters, followed by type 2 diabetes in most clusters.

**Fig. 3:**
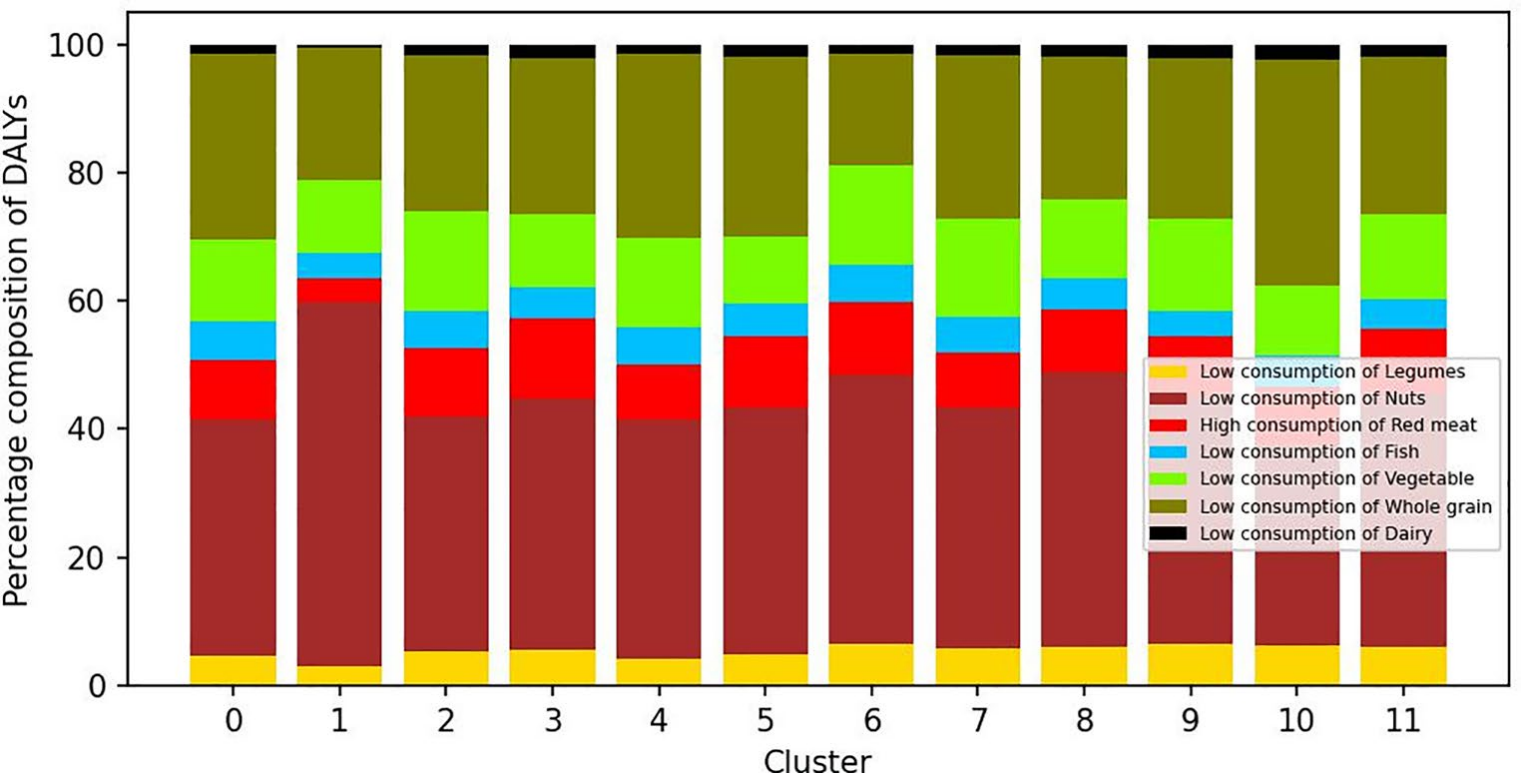
Contribution of seven dietary risk factors to disease burden in 12 clusters of the Danish population disability adjusted life years (DALYs) per 100,000 inhabitants

The relative contribution of different risk factors to the overall burden of disease in each cluster also varied (Fig. 3). Across clusters, the risk factor contributing the most to the burden was too low consumption of tree nuts and peanuts (between 31.2% and 56.8%), followed by too low consumption of whole grains (between 17.48% and 35.31%). In contrast, too low consumption of dairy contributed the lowest to the combined burden across all the clusters (between 0.67% and 2.36%). The contribution of too low consumption of tree nuts and peanuts to the overall burden was highest in cluster 1. Too high consumption of meat contributed between 3.5 and 12.5% of the DALYs in different clusters and was highest in cluster 3. The contribution of risk factors such as too low consumption of fish, legumes, vegetables and fruits were relatively similar across risk factors. Table 3 presents the estimated burden of disease per 100,000 inhabitants attributable to each risk factor in the 12 clusters.

Some of the more outstanding differences in overall burden of disease and contribution of different risk factors to the burden across clusters were accompanied by differences in demographics, socioeconomic or lifestyle characteristics of the individuals in those groups. Cluster 1 was largely constituted of children; it had the lowest average age (8 years old) and the largest percentage of the population below 18 years old. Figures 1 and 2 show that the burden of disease in this cluster was the lowest, and that the too low consumption of dairy and legumes, and too high consumption of red meat were of lower importance. On the other hand, the five clusters with the highest overall disease burden (cluster 8,9,7,3 and 11) had the highest average age (from 48 to 59 years old) and were comprised of mostly adults. The five clusters with the highest burden have an average BMI over 25 (overweight) and tend to live in areas with a smaller population. Clusters 8 and 9 have the lower levels of *low physical activity* and the highest levels of *high physical activity*, and slightly higher percentages of non-smokers than the average. Across the six other clusters with normal BMI, they tend to live in larger municipalities, with different higher education levels between them (3-fold difference) and they have the highest level of very high physical activity. Clusters characterized by living in urban areas, higher education and high physical activity (clusters 5 and 6) carried the lowest burden.

**Table 3:** Estimated disability adjusted life years (DALYs) attributable to seven dietary risk factors in 12 clusters of the Danish population, 2019 (DALY per 100,000 inhabitants, population mean (µ) and standard deviation (σ)). IHD: ischemic heart disease. CVD: cardiovascular disease. T2D: type II diabetes. CRC: colorectal cancer

Cluster			0	1	2	3	4	5	6	7	8	9	10	11
Low consumption of legumes	IHD	µ	9.1	0.4	17.1	78.1	8.0	18.0	42.9	100.6	152.5	122.9	59.6	108.1
		σ	4.7	0.2	9.0	41.6	4.3	10.2	23.4	53.3	81.1	66.4	34.0	58.1
Low consumption of peanuts and tree nuts	IHD	µ	40.4	1.7	72.7	358.6	37.0	85.4	177.9	437.2	696.1	494.1	192.5	465.8
		σ	5.8	0.2	11.3	51.2	5.3	13.0	30.1	66.5	99.4	85.3	40.9	73.3
	CVD	µ	73.2	7.3	119.5	543.0	70.7	142.2	273.9	668.3	1,067.4	742.9	297.0	700.5
		σ	19.6	2.0	34.2	144.5	18.7	40.0	84.1	188.4	284.1	230.2	108.3	203.1
High consumption of red meat	CRC	µ	6.1	0.2	12.1	61.4	5.4	13.7	28.5	61.9	99.1	72.8	34.7	73.4
		σ	0.8	0.0	1.6	7.1	0.7	1.8	3.9	8.8	13.3	11.0	5.4	9.9
	T2D	µ	12.2	0.3	23.1	113.8	11.1	27.6	46.0	88.2	142.1	100.9	52.4	110.2
		σ	0.6	0.0	1.1	4.6	0.6	1.3	2.8	5.5	7.6	6.4	3.5	6.0
Low consumption of fish	STROKE	µ	5.6	0.3	8.2	27.9	5.3	8.6	16.0	40.3	49.2	30.4	19.0	33.3
		σ	3.6	0.2	5.3	19.3	3.5	6.0	11.4	27.9	35.8	23.6	14.3	24.4
	IHD	µ	6.3	0.3	10.3	40.6	5.5	10.7	22.5	57.8	71.5	45.6	27.6	49.4
		σ	3.6	0.1	6.1	25.5	3.2	6.8	14.5	36.1	47.1	32.1	18.9	32.8
Low consumption of dairy	CRC	µ	1.4	0.0	2.7	16.4	1.3	3.5	4.2	16.3	24.9	23.0	11.7	19.0
		σ	0.4	0.0	0.6	3.8	0.3	1.0	1.1	4.3	6.7	6.6	3.2	5.2
	HYPERTENSION	µ	0.0	0.0	0.1	0.5	0.0	0.1	0.1	0.5	0.8	0.7	0.3	0.6
		σ	0.0	0.0	0.0	0.1	0.0	0.0	0.0	0.2	0.3	0.2	0.1	0.2
	T2D	µ	1.6	0.1	2.8	13.1	1.6	3.8	4.9	14.0	20.1	19.1	10.4	16.2
		σ	1.1	0.0	1.8	8.2	1.0	2.6	3.4	9.5	13.5	13.1	7.1	10.9
Low consumption of vegetables	STROKE	µ	9.7	0.6	17.8	54.1	10.5	14.8	34.6	89.8	106.5	91.4	36.8	78.0
		σ	5.2	0.3	9.5	29.9	5.6	9.1	20.1	48.1	60.3	52.4	21.8	43.8
	IHD	µ	12.3	0.7	26.0	83.2	12.5	18.9	52.5	145.9	162.4	146.2	52.2	123.2
		σ	4.7	0.2	9.7	32.9	4.6	8.9	22.7	54.8	65.9	60.9	22.6	50.0
	CRC	µ	3.3	0.2	7.0	21.3	3.4	5.1	13.7	37.0	40.7	37.5	13.5	31.6
		σ	1.4	0.1	2.9	9.4	1.4	2.7	6.6	15.6	18.5	17.4	6.5	14.2
Low consumption of whole grain	IHD	µ	11.8	0.3	17.2	83.6	10.5	22.5	26.4	112.7	141.8	120.7	82.8	110.1
		σ	3.0	0.1	4.6	23.4	2.7	6.0	8.6	32.2	41.8	36.9	22.1	32.4
	T2D	µ	19.4	0.6	27.8	108.0	18.9	36.1	41.3	138.8	166.4	150.2	107.1	138.2
		σ	5.9	0.2	9.4	37.3	6.0	11.9	17.9	48.2	61.2	56.2	35.0	50.2
	CRC	µ	5.3	0.1	7.8	35.3	4.8	10.0	11.4	46.9	58.4	50.7	35.1	46.3
		σ	1.9	0.1	3.0	14.5	1.8	3.9	5.5	19.6	25.1	22.9	13.8	19.8
	CVD	µ	33.0	1.9	43.6	193.9	31.0	57.7	61.7	264.3	331.9	277.2	193.7	253.5
		σ	9.3	0.7	13.0	61.5	9.0	17.2	22.9	85.3	110.3	96.3	58.8	83.6
Total		µ	209	14.18	326.73	1429.5	206.92	394.05	689.68	1768.22	2622.34	1971.02	959.8	1834.26

### Mean food consumption and comparison with dietary recommendations

**Fig. 4:**
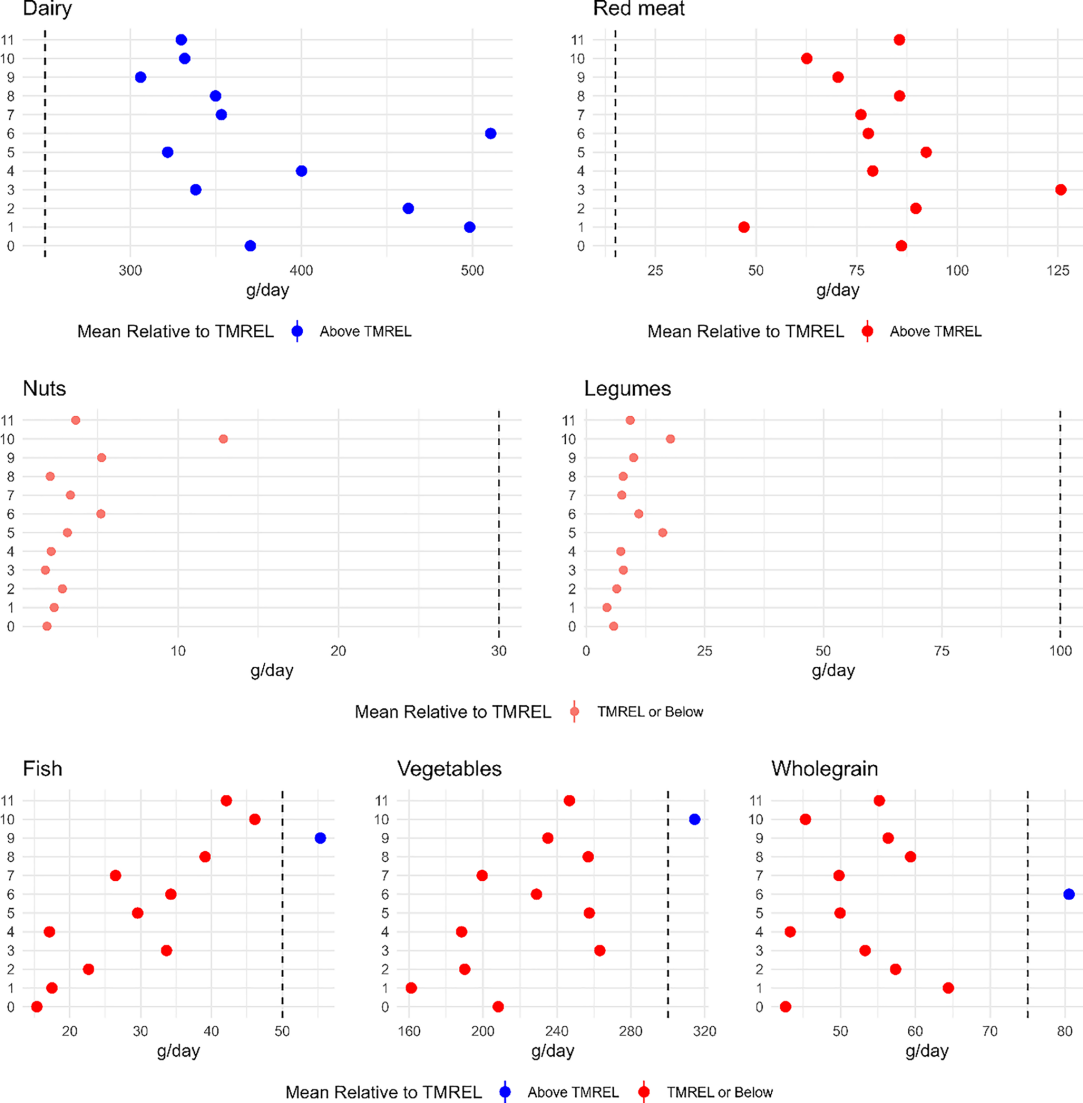
Mean consumption of food groups in their respective clusters. The red circles indicate consumption below TMREL, and blue circle indicates consumption above TMREL

The burden of disease and contribution of risk factors to total DALY reflected the consumption of each food category in each cluster, how it compares with the recommended intake of each food category (TMREL), and of the DALY envelopes representable of the age and gender structure of each cluster. Figure 4 shows that clusters 0, 1, 2, 3 and 4 had the lowest mean consumption of fish, fruit and vegetables, tree nuts and peanuts, and legumes and were furthest away from the recommended intake of those food groups (i.e., the TMREL). Clusters 0, 3, 8, and 11 had the highest consumption of red meat, and clusters 9, 5 and 3 had the lowest consumption of dairy. All clusters had an average consumption of dairy above the recommended intake. In contrast, consumption adhering to the recommended minimum intake of vegetables, fish, and whole grain was only observed in three (different) clusters (Fig. 4). The above observations are reflective of the distinct characteristics. For example, cluster 10 grouped individuals that are female, highly educated, older, non-smokers, exercise frequently and live in municipalities of large population size. They consume the least amount of red meat and the most amount of nuts, pulses and vegetables among the clusters. This is reflected in the burden contribution from the respective food groups, especially nuts, which has the lowest contribution in cluster 10 compared to other clusters. It is also the only cluster that has the consumption of vegetables above TMREL. On the other hand, cluster 3 grouped individuals living in sparsely populated municipalities have a lower education and low physical activity levels. They also tend to smoke more, and majority comprised of men. This group has the highest consumption of red meat (as high as 8 times the TMREL and 2 times the consumption of cluster 10) and the lowest consumption of nuts (as low as 10 times lower than the TMREL and 7 times lower than cluster 10). These consumption patterns are not surprising, as cluster 3 individuals tend to have a more traditional meat heavy diet compared to their urban and liberal counterparts from cluster 10. Surprisingly, consumption of dairy is above the TMREL among all the clusters, which reflects in the very low burden, and suggests that it remains a popular food choice irrespective of the socio-economic characteristics.

## Discussion and conclusion

We estimated the burden of disease attributable to seven dietary risk factors in 12 subgroups of the Danish population in 2019. The seven risk factors were associated with five NCDs that are leading causes of the loss of healthy life years in Denmark. Our results showed that the burden of sub-optimal diets is unevenly distributed across population groups that have similar characteristics in terms of diet, lifestyle and socioeconomics. These groupings go beyond the traditional categorization of the population in terms of age, sex and geography, and pinpoint to groups of individuals in which targeted interventions could have a larger impact in terms of reducing disease burden.

In general, clusters with individuals living in urban areas, with a high level of education, moderate or high physical activity habits and generally considered “healthy” dietary habits (e.g. with high consumption of fruit and vegetables, cereal-based foods and fish) carried a lower burden of disease due to the seven risk factors (clusters 0, 2 and 4). In contrast, the burden of disease in clusters 8, 9, 11, and 7 was highest, largely explained by low consumption of nuts and whole grain, of which individuals in these clusters had a mean consumption farthest away from the recommended intakes. Individuals in these clusters typically lived in rural areas, had a lower level of education than the general population.

Cluster 1, which had the lowest estimated DALY/100,000 inhabitants, had a high proportion of children and low mean age. The population structure explains the low burden of disease, both because of dietary habits and the low incidence of NCDs, which are largely lifestyle chronic diseases that develop later in life.

The contribution of low consumption of tree nuts and whole grains to the overall disease burden was high across clusters. The high disease burden is partly explained by the low consumption of these two food groups in the population compared to the recommended intake. Large gaps between current and optimal intake of nuts and whole grains have been observed in studies of other countries [1, 11]. In contrast, dairy had a relatively low contribution to the burden of disease, reflecting the high consumption of dairy in all clusters. Thus, when the intake is above the TMREL in all the clusters, the risk factor is not contributing to the burden (i.e. the intake of dairy is high enough). That dairy still contributes slightly to the burden due to the stochastic nature of the model allowing for sampling a mean intake of a given cluster below the TMREL.

Herrera et al. 2021 [6] published the burden of disease of dietary exposure to three heavy metals (MeHg, iAs and Cd ) in the same population clusters in Denmark and observed that clusters 5, 6 and 10 had the highest burden caused by these chemicals and were composed of individuals with higher education and physical activity, were mainly non-smokers and lived in urban areas, and had a higher adherence with dietary patterns considered healthy. We observed that the ranking of the clusters with highest burden due to the dietary risk factors was opposite. For example, clusters 3 and 8 had a lower burden due to chemical exposures, but a higher burden due to the dietary risk factors. Clusters 3 and 8 were characterized by lower education, rural areas, smoking and low physical activity. However, the magnitude of the burden caused by exposure to the chemicals included in the study is considerably lower than the burden posed by exposure to the dietary risk factors, for example in cluster 8, burden due to the chemicals is below 10 DALY/100,000 whereas burden due to dietary risk factors is 2,622 DALY/100,000.

While it is well known that foods can be contaminated with chemicals that may lead to a variety of adverse health effects [11, 25] originating from e.g. environmental pollution, food packaging and food processing, and scientific and policy efforts are established to ensure that foods are safe for human consumption, public health policies do not often tackle nutrition and food safety considerations together. Across the clusters, whole grain is under-consumed compared to the recommended intake, except for cluster 6, which happens to have the highest burden due to exposure to MeHg, iAs and Cd and low burden on low consumption of whole grains. These results show that strategies that promote dietary changes for public health impacts should consider both nutritional and food safety aspects of foods. Studies that assess the combined risks and beneficial impacts of foods, also known as risk-benefit assessments, consider nutritional risks and benefits and food safety risks in one single approach, but typically do that at a single food level and compare the impacts of different hypothetical consumption scenarios [26]. Furthermore, dietary patterns and consequently dietary exposure to chemicals are largely influenced by socio-economic factors, such as education, income, and culture [4, 5] and their impact on the overall burden of disease is seldom investigated.

The choice of a suitable distribution plays an important role in the accuracy and the precision of the estimates. This is often a practical choice, based on empirical data and domain knowledge. In our model, we applied a normal distribution to the parameters of food intake and DALY envelopes, whereas the dose-response relationship was modelled on a log-normal distribution, which was deemed suitable due to the perceived skewness towards the lower uncertainty bound. For modelling consumption, an alternative would be to positive value distributions like log-normal or gamma. This should be done with caution due to the presence of zero consumption individuals for certain food groups, as log transformation yields negative infinity values. One way to mitigate this would be to divide the consumption into two parts, namely zero and non-zero consumption and model them with a binomial distribution first and then a log-normal distribution for the non-zero consumption. Due to the relatively large variation of data samples across clusters, we deemed the normal distribution fit as a safe and simpler choice. When applying the normal distribution, we clipped all sampled negative consumption to zero during the sampling step of the Monte Carlo process.

The choice of using a log-linear association to model the relative risk and exposure is an accepted gold standard in literature. Many epidemiological studies suggest that risk often follows a log-linear pattern for dietary factors. They are also mathematically simple and provide a convenient way to represent risks while avoiding negative values and maintaining interpretability. There are some situations where non-linear associations matter, namely cases where risks only emerge beyond a certain exposure level. A possible improvement could be to test alternative models like a piecewise or sigmoid function.

By assigning DALY envelopes to each individual, we have introduced uncertainty in the overall DALY estimates that were not quantified. As DALY was sampled from a sub-population distribution, each individual was assigned a slightly different DALY estimate in each iteration. We assessed the impact of this process by running multiple iterations for some of the risk factor– health outcome pairs with the same parameters and found results did not vary substantially between iterations. The standard deviation of the DALY/100k sampling distribution varied substantially for different outcome-risk factor combinations. These differences may be explained by the differences in population structures between clusters (in terms of age and sex), which is reflected in the consumption distributions in each cluster.

We quantified the uncertainty around our estimates by applying a probabilistic approach with a sample size of 10,000, which we assessed to be a good balance between convergence of the model computational resources needed.

In addition to modelling uncertainties, data constraints led to uncertainties in the results. Identification of the health outcomes associated with the risk factors was based on the review of scientific evidence, and some health outcomes were not included due to low weight of evidence. We applied the Danish FBDGs to define the TMREL of food groups (except for red meat), which are aligned with the latest updates of Nordic Nutritional Recommendations 2023 (NNR 2023) [27]. The maximum consumption of meat recommended by Danish FBDGs includes all types of meat (beef, pork, poultry, and lamb), while the health outcomes associated with meat consumption that we selected have been associated with the consumption of red meat only; thus we assumed the consumption of red meat alone as defined by Lassen et al. in a Danish-adapted healthy plant-based diet [14]. Although the Danish official dietary guidelines are aligned with the NNR 2023, there are some differences between them in terms of the recommended intake for some of the food groups. For example, NNR 2023 gives a general recommendation of including legumes in the diet, while Danish guidelines give clear instructions to consume about 100 g of legumes per day. Recommendations for consumption of dairy are also slightly different: NNR 2023 recommends 350–500 g of dairy daily, while the Danish FBDG recommend 250–350 g of dairy and 20 g of cheese (or additional 100 g of dairy) daily. Differences are summarized in Supplementary item S5.

The literature review to identify the health outcomes associated with dietary risk factors for our study was conducted in 2021 and does not include the most recent sources of evidence such as the latest systematic reviews performed for NNR 2023, such as [28–30]. This is a limitation of our study. The differences between the health outcomes associated with the intake of seven food groups selected for our study and in NNR 2023 are summarized in the Supplementary item S5.

Additionally, food consumption data were available from the Danish Survey on Diet and Physical Activity (DANSDA) 2011-13, which has by now been collected over a decade ago. We assumed that food consumption patterns have not changed substantially in the population during this period. This is a limitation, as food sales data suggest that for example consumption of meat and milk have decreased and of nuts have increased in Denmark [31]. When more recent data becomes available, our model can be updated to reflect the newest evidence available and current dietary patterns in the population.

The above data also considered the instances of underreporting of energy intakes using Goldebrg’s cutoff and the validation of seven-day food diary among adults showed that energy intake on average was underestimated by 12–16% compared with energy expenditure which we have not included in the design of the models.

In conclusion, our results showed that the burden of sub-optimal diets is unevenly distributed across population groups that have similar characteristics in terms of diet, lifestyle and socioeconomics. This approach can be useful to inform public health policies that are more targeted towards sub-groups in the population at higher risk.

## Electronic supplementary material

Below is the link to the electronic supplementary material.

Supplementary Material 1
