# Supplementary material for: Burden of disease of seven dietary risk factors in population groups with similar lifestyle patterns in Denmark
Source: Eur J Nutr. 2025 Jun 2;64(5):201. doi: 10.1007/s00394-025-03715-9 (PMC12130065; doi:10.1007/s00394-025-03715-9)
Supplement: Supplementary file 1 — Supplementary Material 1 [file 394_2025_3715_MOESM1_ESM.docx]

**Supplementary material**

**Burden of disease of seven dietary risk factors in population groups with similar lifestyle patterns in Denmark**

Sayantan Sengupta^1^; Sofie T. Thomsen^1^, Aleksandra Davydova^1^; Hernan G. Redondo^1^, Lea S. Jakobsen^1^, Sara M. Pires^1^

^1^Risk-Benefit Research Group, National Food Institute, Technical University of Denmark, Lyngby, Denmark

S 1*. Pipeline of the algorithm for n iterations of probabilistic estimation of burden of food group x.*

| **Probabilistic estimation of Burden of health outcome *j* due to food item *x*** | | |
| --- | --- | --- |
|  | ***Intake Distribution:*** *Normal (mean intake, standard deviation intake)* | |
|  | ***Relative Risk Distribution:*** *Log Normal (log mean, Log standard deviation)* | |
|  | ***Dose Response Function:*** *Mixed linear model (relative risk)* | |
|  | ***DALY Distribution:*** *Normal (mean DALY, standard deviation DALY)* | |
| **1** | $For iteration i from 1 to n:$ | |
| **2** |  | *S = Random sample from* ***Intake Distribution*** |
| **3** |  | *S*$'$*= TMREL for food item* ***x*** |
| **4** |  | *R= Random sample from* ***Relative Risk Distribution*** |
| **5** |  | *D= random sample from* ***DALY Distribution*** |
| **6** |  | *Relative Risk⁽ ⁱ ⁾= Mixed linear model (R)* |
| **7** |  | $Impact Fraction⁽ ⁱ ⁾ =\frac{Relative Risk⁽ ⁱ ⁾(S) - Relative Risk⁽ ⁱ ⁾(S’)}{Relative Risk⁽ ⁱ ⁾(S)}$ |
| **8** |  | *Final Burden*$⁽ ⁱ ⁾$ *=* $Impact Fraction⁽ ⁱ ⁾ \times D$ |
| **9** | ***end*** | |

S 2. *Estimated population attributable factors (PAF) for selected dietary risk factors-health outcome pairs in 11 clusters of the Danish population. T2D - type 2 diabetes, CRC - colorectal cancer, CC - colon cancer, IHD - ischemic heart disease, CVD – cardiovascular disease.*

| Cluster | | | 0 | 1 | 2 | 3 | 4 | 5 | 6 | 7 | 8 | 9 | 10 | 11 |
| --- | --- | --- | --- | --- | --- | --- | --- | --- | --- | --- | --- | --- | --- | --- |
| Too low consumption of Legumes | IHD | µ | 0.07 | 0.07 | 0.07 | 0.07 | 0.07 | 0.06 | 0.07 | 0.07 | 0.07 | 0.07 | 0.06 | 0.07 |
|  |  | σ | 0.04 | 0.04 | 0.04 | 0.04 | 0.04 | 0.04 | 0.04 | 0.04 | 0.04 | 0.04 | 0.04 | 0.04 |
| Low consumption of Nuts | IHD | µ | 0.27 | 0.27 | 0.26 | 0.28 | 0.28 | 0.26 | 0.24 | 0.26 | 0.28 | 0.24 | 0.17 | 0.26 |
|  |  | σ | 0.04 | 0.04 | 0.04 | 0.04 | 0.04 | 0.04 | 0.05 | 0.04 | 0.04 | 0.05 | 0.04 | 0.04 |
|  | CVD | µ | 0.21 | 0.21 | 0.20 | 0.21 | 0.21 | 0.20 | 0.19 | 0.20 | 0.21 | 0.18 | 0.14 | 0.20 |
|  |  | σ | 0.04 | 0.04 | 0.04 | 0.04 | 0.04 | 0.04 | 0.05 | 0.05 | 0.04 | 0.04 | 0.04 | 0.05 |
| High consumption of Meat | T2D | µ | 0.10 | 0.05 | 0.11 | 0.14 | 0.09 | 0.11 | 0.09 | 0.09 | 0.10 | 0.08 | 0.07 | 0.10 |
|  |  | σ | 0.01 | 0.01 | 0.01 | 0.01 | 0.01 | 0.01 | 0.01 | 0.01 | 0.01 | 0.01 | 0.01 | 0.01 |
|  | CRC | µ | 0.06 | 0.04 | 0.07 | 0.08 | 0.06 | 0.06 | 0.06 | 0.06 | 0.06 | 0.06 | 0.05 | 0.06 |
|  |  | σ | 0.01 | 0.01 | 0.01 | 0.01 | 0.01 | 0.01 | 0.01 | 0.01 | 0.01 | 0.01 | 0.01 | 0.01 |
| Low consumption of Fish | STROKE | µ | 0.05 | 0.05 | 0.04 | 0.04 | 0.05 | 0.04 | 0.04 | 0.04 | 0.03 | 0.03 | 0.03 | 0.03 |
|  |  | σ | 0.03 | 0.03 | 0.03 | 0.02 | 0.03 | 0.02 | 0.02 | 0.03 | 0.02 | 0.02 | 0.02 | 0.02 |
|  | IHD | µ | 0.04 | 0.04 | 0.04 | 0.03 | 0.04 | 0.03 | 0.03 | 0.03 | 0.03 | 0.02 | 0.02 | 0.03 |
|  |  | σ | 0.02 | 0.02 | 0.02 | 0.02 | 0.02 | 0.02 | 0.02 | 0.02 | 0.02 | 0.01 | 0.02 | 0.02 |
| Low consumption of Dairy | CRC | µ | 0.01 | 0.01 | 0.01 | 0.02 | 0.01 | 0.02 | 0.01 | 0.02 | 0.02 | 0.02 | 0.02 | 0.02 |
|  |  | σ | 0.00 | 0.00 | 0.00 | 0.00 | 0.00 | 0.00 | 0.00 | 0.00 | 0.00 | 0.01 | 0.00 | 0.00 |
|  | HYPERTENSION | µ | 0.01 | 0.01 | 0.01 | 0.01 | 0.01 | 0.01 | 0.01 | 0.01 | 0.01 | 0.01 | 0.01 | 0.01 |
|  |  | σ | 0.00 | 0.00 | 0.00 | 0.00 | 0.00 | 0.00 | 0.00 | 0.00 | 0.00 | 0.00 | 0.00 | 0.00 |
|  | T2D | µ | 0.01 | 0.01 | 0.01 | 0.02 | 0.01 | 0.01 | 0.01 | 0.01 | 0.01 | 0.02 | 0.01 | 0.01 |
|  |  | σ | 0.01 | 0.01 | 0.01 | 0.01 | 0.01 | 0.01 | 0.01 | 0.01 | 0.01 | 0.01 | 0.01 | 0.01 |
| Low consumption of Vegetable | STROKE | µ | 0.08 | 0.10 | 0.09 | 0.07 | 0.09 | 0.07 | 0.07 | 0.09 | 0.07 | 0.07 | 0.06 | 0.07 |
|  |  | σ | 0.05 | 0.06 | 0.06 | 0.05 | 0.06 | 0.05 | 0.05 | 0.06 | 0.05 | 0.05 | 0.04 | 0.05 |
|  | IHD | µ | 0.08 | 0.11 | 0.09 | 0.06 | 0.09 | 0.06 | 0.07 | 0.09 | 0.07 | 0.07 | 0.05 | 0.07 |
|  |  | σ | 0.03 | 0.04 | 0.04 | 0.03 | 0.04 | 0.03 | 0.03 | 0.04 | 0.03 | 0.03 | 0.02 | 0.03 |
|  | CRC | µ | 0.03 | 0.04 | 0.03 | 0.02 | 0.03 | 0.02 | 0.03 | 0.03 | 0.02 | 0.03 | 0.02 | 0.02 |
|  |  | σ | 0.01 | 0.02 | 0.01 | 0.01 | 0.02 | 0.01 | 0.01 | 0.01 | 0.01 | 0.01 | 0.01 | 0.01 |
| Low consumption of Whole grain | IHD | µ | 0.08 | 0.05 | 0.06 | 0.06 | 0.08 | 0.07 | 0.03 | 0.07 | 0.05 | 0.06 | 0.07 | 0.06 |
|  |  | σ | 0.02 | 0.02 | 0.02 | 0.02 | 0.02 | 0.02 | 0.01 | 0.02 | 0.02 | 0.02 | 0.02 | 0.02 |
|  | T2D | µ | 0.13 | 0.08 | 0.10 | 0.11 | 0.13 | 0.11 | 0.06 | 0.11 | 0.09 | 0.10 | 0.12 | 0.10 |
|  |  | σ | 0.05 | 0.04 | 0.04 | 0.05 | 0.05 | 0.05 | 0.04 | 0.05 | 0.05 | 0.05 | 0.05 | 0.05 |
|  | CRC | µ | 0.06 | 0.03 | 0.04 | 0.05 | 0.06 | 0.05 | 0.03 | 0.05 | 0.04 | 0.04 | 0.05 | 0.04 |
|  |  | σ | 0.02 | 0.02 | 0.02 | 0.02 | 0.02 | 0.02 | 0.01 | 0.02 | 0.02 | 0.02 | 0.02 | 0.02 |
|  | CVD | µ | 0.09 | 0.05 | 0.07 | 0.07 | 0.09 | 0.08 | 0.04 | 0.07 | 0.06 | 0.06 | 0.08 | 0.07 |
|  |  | σ | 0.03 | 0.02 | 0.02 | 0.03 | 0.03 | 0.03 | 0.02 | 0.03 | 0.02 | 0.03 | 0.03 | 0.02 |

S 3. Moving average of combined DALY/100k of 12 clusters for all risk factor-health outcome pairs upto 50 monte carlo iterations.

| 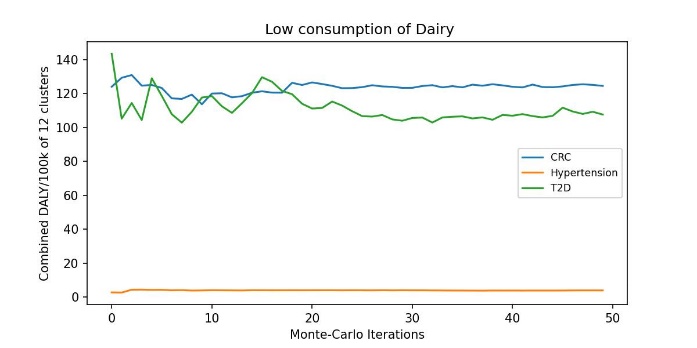 | 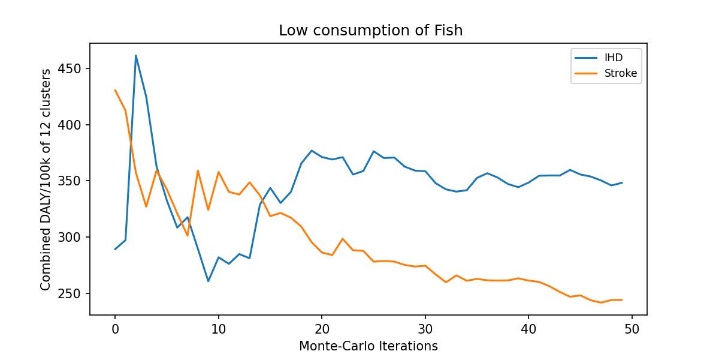 |
| --- | --- |
| 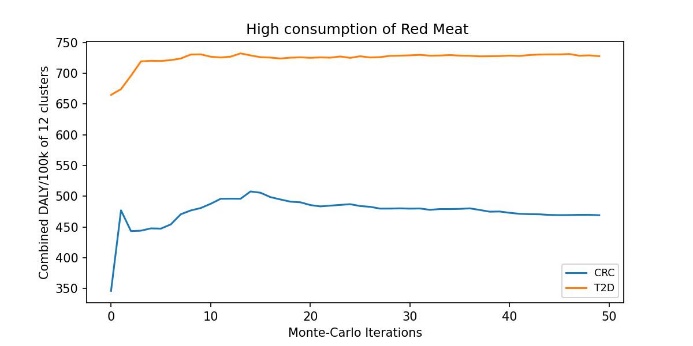 | 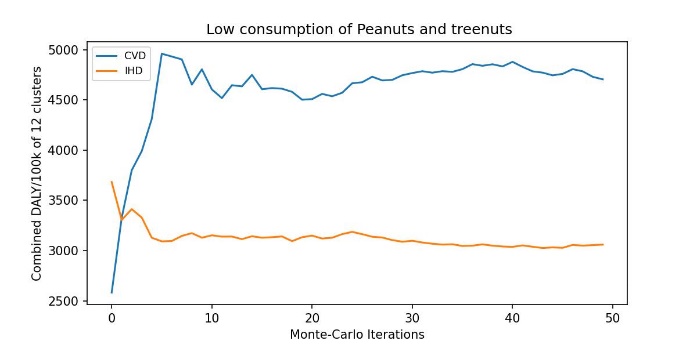 |
| 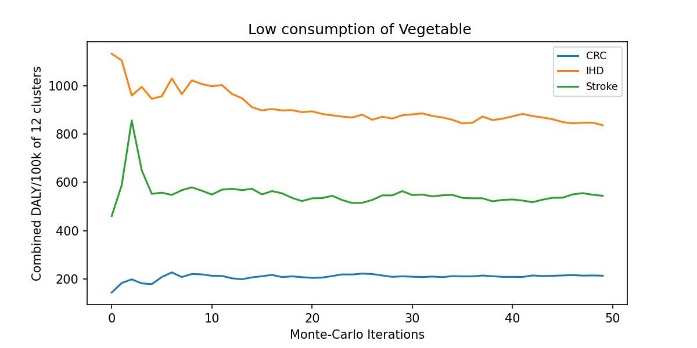 | 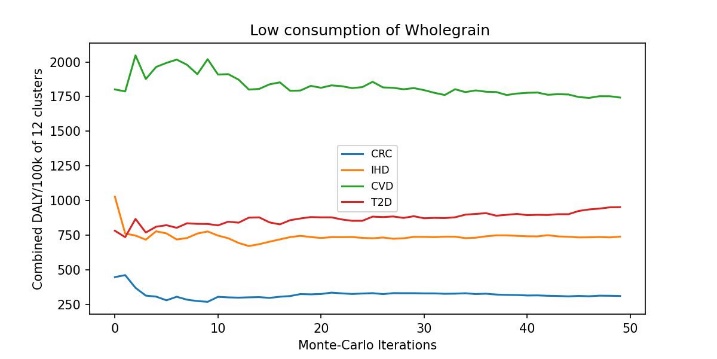 |
| 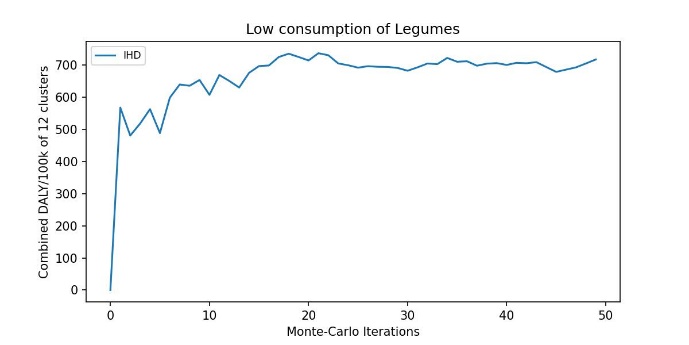 | |

S 4. Mean intake(gm/day) of selected seven food groups for each cluster along with their respective TMREL.

| ***Cluster*** | **Dairy** | **Fish** | **Meat** | **Nuts** | **Pulses** | **Vegetable** | **Whole grain** |
| --- | --- | --- | --- | --- | --- | --- | --- |
| 0 | 370.13 | 15.35 | 86.11 | 1.84 | 5.77 | 208.24 | 42.66 |
| 1 | 498.3 | 17.48 | 46.98 | 2.29 | 4.33 | 161.15 | 64.41 |
| 2 | 462.44 | 22.65 | 89.7 | 2.8 | 6.41 | 190.1 | 57.35 |
| 3 | 338.11 | 33.65 | 125.74 | 1.74 | 7.82 | 263.17 | 53.29 |
| 4 | 400.02 | 17.13 | 78.97 | 2.1 | 7.26 | 188.35 | 43.28 |
| 5 | 321.73 | 29.57 | 92.26 | 3.11 | 16.1 | 257.49 | 49.95 |
| 6 | 510.53 | 34.27 | 77.87 | 5.2 | 11.08 | 228.92 | 80.53 |
| 7 | 353.16 | 26.45 | 76.03 | 3.3 | 7.49 | 199.55 | 49.79 |
| 8 | 349.75 | 39.1 | 85.63 | 2.04 | 7.76 | 256.9 | 59.36 |
| 9 | 305.9 | 55.37 | 70.35 | 5.24 | 9.96 | 235.07 | 56.37 |
| 10 | 331.68 | 46.12 | 62.6 | 12.83 | 17.75 | 314.49 | 45.32 |
| 11 | 329.67 | 42.1 | 85.61 | 3.63 | 9.25 | 246.74 | 55.18 |
| ***TMREL*** | *250* | *50* | *15* | *30* | *100* | *300* | *75* |

S 5. Comparison of the different sources of recommendations for the intake of seven food groups, and health effects associated with the intake of the food groups. ↓ represents reduction in risk of the health outcome and ↑ represents increase in risk of the health outcome.

| **Food group** | **Associated health outcomes (selected in our study, updates 2021)** | **TMREL (selected for our paper, updates 2021), g/day** | **Recommended intake by NNR 2023** | **Associated health outcomes (updates for NNR 2023)** | **Recommended intake by Danish FBDG** |
| --- | --- | --- | --- | --- | --- |
| Nuts | ↓CVD, IHD | 30 | 20-30 g/day | ↓CVD, CHD; suggestive for stroke, insuffisient for T2D and others | 30 g/day |
| Legumes | ↓IHD | 100 | Recommended to include in the diet | ↓CVD, T2D, cancer, mortality | 100 g/day |
| Fish | ↓IHD, Stroke | 50 | 350 g/week (including at least 200 g of fatty fish) | ↓CHD, MI, Stroke, ACM | 350 g/week (including at least 200 g of fatty fish) |
| Dairy | ↓T2D, Hypertension | 250 | 350-500 g/day | ↓CVD, CRC, T2D, ACM, Low fat dairy ↓Obesity | 250-350 g/day and 20 g of cheese (or extra 100 ml of dairy) |
| Whole grain | ↓T2D, CRC, IHD, CVD | 75 | At least 90 g/day | ↓CVD, CRC, T2D, ACM, CHD | 90 g/day* |
| Vegetables | ↓Stroke, CRC, IHD | 300 | 500-800 g /day of vegetables, fruits and berries (potato is not included) | ↓CVD, BC, CRC, BW, aerodigestive cancers | 600 g /day of vegetables, fruits and berries (potato is not included) |
| Red meat | ↑CRC, CC, T2D | 15 | Max. 350 g/week | ↑CRC, CVD | Max. 350 g/week of white and red meat, limit beef and lamb |

*Was updated recently based on NNR 2023 (we use 75 g/day in our study)
